# International Nurse Leaders' Journeys of Engagement, Belonging, and Growth in Professional Nursing Organizations: A Qualitative Study

**DOI:** 10.1111/jnu.70125

**Published:** 2026-08-25

**Authors:** Jihane Frangieh, Edmund J. Walsh, Kay Kennedy, Kim Crawford, Cindy Zellefrow, Beverly Hancock, Mary A. Dolansky

**Affiliations:** ^1^ School of Nursing Johns Hopkins University Baltimore Maryland USA; ^2^ Arthur Labatt Family School of Nursing University of Western Ontario London Ontario Canada; ^3^ uLeadership Atlanta Georgia USA; ^4^ Tanner Health School of Nursing University of West Georgia Carrollton Georgia USA; ^5^ College of Nursing The Ohio State University Columbus Ohio USA; ^6^ College of Nursing Rush University Chicago Illinois USA; ^7^ Frances Payne Bolton School of Nursing Case Western Reserve University Cleveland Ohio USA

**Keywords:** belonging, global engagement, international collaboration, international membership, member engagement, nursing leadership, professional nursing organizations

## Abstract

**Introduction:**

Professional nursing organizations play a central role in connecting nurse leaders across borders, advancing leadership science, and addressing shared challenges, including workforce sustainability, burnout, and policy influence. Little is known about how specialized professional nursing organizations with a primarily national membership base can build and sustain meaningful international membership. Barriers to and facilitators of international membership in professional nursing leadership organizations have received limited attention in the literature, particularly from the perspectives of international members themselves. The aims of the study are to explore the perspectives of international nurse leaders enrolled in the Association for Leadership Science in Nursing International Ambassador Program on what motivates and sustains their engagement, the leadership‐related benefits they seek, the organizational supports that would make engagement more feasible, and the contributions they bring as members of an international nursing leadership community.

**Design:**

A qualitative descriptive design was used.

**Methods:**

Data were collected using a Zoom‐based focus group with five of 11 nurse leader ambassadors enrolled in the program. Data were analyzed using Braun and Clarke's six‐phase reflexive thematic analysis. Trustworthiness was supported through investigator triangulation, peer debriefing, an audit trail, and reflexive review of researcher perspectives.

**Results:**

Four themes were developed: (1) *reciprocal exchange*, in which members positioned themselves as contributors as well as recipients; (2) *engaging on our own terms*, framing access and context‐sensitivity as preconditions of membership; (3) *belonging as more than membership*, distinguishing formal affiliation from felt inclusion in a community of nurse leaders; and (4) *growth through sustained international relationships,* in which professional growth depends on ongoing cross‐border connection rather than time‐bound programs.

**Conclusion:**

Findings suggest that international nursing leadership organizations should build infrastructure for reciprocal contribution, equitable access, substantive belonging, and sustained cross‐border relationships to engage and retain global members.

**Clinical Relevance:**

Engagement in global nursing communities facilitates the exchange of knowledge, best practices, and innovations across healthcare settings. These opportunities can strengthen leadership development, support evidence‐based practice, and ultimately improve patient outcomes.

## 1 Introduction

Nursing leadership scientists worldwide are committed to promoting leadership development and addressing key challenges affecting the profession, including unhealthy work environments and threats to workforce sustainability (Chen et al. 2024). Leaders around the globe share concerns related to burnout, recruiting and retaining nurses (Adhikari and Smith 2023), and operating within fiscal constraints (World Bank 2026), among many others. While these shared concerns may present differently, vary in intensity, arise from different antecedents, and be shaped by different cultural contexts, they remain common across countries. Opportunities to advance nursing leadership practice, education, and research internationally should therefore be explored (Cummings et al. 2018).

Nursing is an interconnected global profession in which strategies implemented in one country can affect another. For instance, a country's nurse recruitment strategy may undermine another's nurse retention strategy (Adhikari and Smith 2023). This interdependence makes connecting leadership scientists from around the world not only valuable but necessary. By sharing ideas, concerns, and resources, and by listening to and learning from others' experiences and perspectives, nursing leadership and the profession can be strengthened.

Professional organizations play a key role in building these connections among nurses in practice and academia. Several long‐established international organizations have devoted significant effort to connecting nurses across borders. For instance, the International Council of Nurses (ICN) represents nurses worldwide, advances the profession, promotes nurses' well‐being, and advocates for health in all policies (ICN n.d.‐b). In addition, Sigma Theta Tau International Honor Society of Nursing (Sigma) has members in many countries and convenes one of the largest international audiences of nurse scholars and leaders (Rosser et al. 2023).

Both organizations have made meaningful contributions to global nursing leadership through structured initiatives. The ICN established the Global Nursing Leadership Institute, a one‐year leadership development program for senior nurse leaders that aims to foster sustained global professional connections, increase global leadership knowledge, support learning across countries, and strengthen leadership capacity (Blaney 2012; ICN n.d.‐a; Mason and Salvage 2021). Similarly, in 2014, Sigma established the Global Leadership Mentoring Community, a one‐year online program (Rosser et al. 2023). In the cohort reported by Rosser et al. (2023), 11 countries were represented, with 17 mentor‐mentee pairs recruited from Sigma's seven global regions. Members of each pair came from different global regions, and participants also had opportunities to meet others from additional regions through group meetings. The program provided valuable mentorship experiences while fostering connections across regions, challenging assumptions about other regions, and increasing awareness of issues experienced in other parts of the world (Rosser et al. 2023).

Such programs, in addition to conference sessions (Gantz et al. 2012) and educational initiatives (Bragadôttir and Potter 2019), illustrate the meaningful influence of activities that bring together nursing leaders from across the globe. Many of these programs share a focus on time‐bound cohort experiences, often lasting approximately one year, that build connections through structured formats. However, less is known about how international engagement can be sustained beyond such programs through ongoing membership in specialty nursing organizations. This gap requires understanding how membership in an international nursing leadership organization can be made more valuable and relevant for international members, including by identifying desired activities and mutually beneficial collaboration opportunities.

Scholars have identified numerous barriers to international collaboration among nursing leaders, including expenses associated with attending in‐person events (Blaney 2012), difficulties obtaining travel visas (Shin et al. 2016), language and time zone barriers (Bragadôttir and Potter 2019), technology limitations (Mason and Salvage 2021), and navigating cultural norms (Gilliss and Milone‐Nuzzo 2025). Facilitators include securing major sponsors and establishing bursary programs (Mason and Salvage 2021) as well as using technology to support connection and collaboration (Buckner et al. 2014). While technology offers benefits, online videoconferencing can lead to fatigue and may not fully replicate the richness of in‐person meetings (Mason and Salvage 2021).

The Association for Leadership Science in Nursing (ALSN), formerly the Council on Graduate Education for Administration in Nursing (CGEAN), was established in 1970 (ALSN n.d.) and is dedicated to advancing nursing leadership science to improve outcomes for nurses, students, patients, and healthcare systems. Although ALSN has historically included members from Canada and other countries, its membership is currently concentrated in the United States, with renewed efforts underway to strengthen international engagement. ALSN currently has approximately 350 members, primarily from practice and academia, including nurse scientists, scholar‐practitioners, and educators committed to shaping the future of healthcare through evidence‐based leadership (ALSN n.d.). The organization offers a wide range of opportunities for engagement and professional development, including annual and virtual conferences, monthly webinars, a mentorship program, research funding opportunities, a speakers bureau, and access to a network of established and emerging leaders (ALSN n.d.).

Building on this foundation and the organization's commitment to global engagement, ALSN launched the International Ambassador Program to deepen and broaden international participation. Designed by a team of academic and nurse leaders, the program fosters meaningful international engagement and creates dedicated space for cross‐border dialogue on shared challenges in nursing leadership, education, research, and practice. The program was launched in September 2025 with 11 nursing leaders from 11 countries and regions in Asia, Europe, the Middle East, North America, and South America. Ambassadors held leadership positions across academia, clinical practice, administration, and research within their national and regional contexts. The cohort consisted primarily of senior nursing leaders, alongside a smaller number of emerging leaders. This one‐year experience involves several initiatives, including the focus group reported in this paper, that aim to better understand (a) priority leadership needs across the globe, (b) how ALSN can provide increased value to international members, and (c) the barriers to accessing membership and participation in ALSN. Barriers to and facilitators of membership in an international nursing leadership organization such as ALSN have received limited attention in the literature.

The purpose of this study is to explore the perspectives of international nurse leaders enrolled in the ALSN International Ambassador Program on what motivates and sustains their engagement with international nursing organizations, the leadership‐related benefits they seek from participation, the organizational supports that would make engagement with international professional nursing organizations more feasible and meaningful, and the contributions they bring as members of an international nursing leadership community.

## 2 Design

A qualitative descriptive study design was used to explore nurse leaders' perspectives on engagement in an international nursing leadership organization.

## 3 Materials and Methods

### 3.1 Participants

All 11 nurse leaders enrolled in the ALSN International Ambassador Program were invited by email to participate; five ambassadors, representing five countries, consented to participate and attended the scheduled focus group. The purposive sampling approach was appropriate given participants' shared involvement in the program and their ability to provide relevant insight into global leadership experiences.

### 3.2 Data Collection Procedures

Institutional Review Board approval was obtained from Case Western Reserve University prior to data collection. To support preparation among a culturally and linguistically diverse group, the guiding focus group questions (see Table 1) were included in the email invitation sent in advance (Halcomb et al. 2007). Data were collected using a Zoom‐based focus group. The session was facilitated by two experienced interviewers: one facilitated the discussion, while the other monitored the Zoom chat, observed group interactions, and asked follow‐up questions (Halcomb et al. 2007; Jayasekara 2012). Additional members of the research team attended as observers.

**TABLE 1:** Guiding focus group questions.

Q1	What motivates you to engage with or maintain membership in international nursing organizations such as Sigma Theta Tau or similar global professional bodies?
Q2	What professional or leadership‐related benefits do you seek from participating in international organizations focused on advancing nursing leadership or leadership science?
Q3	What organizational structures, resources, or support would make it easier for you to engage with ALSN as an international member?
Q4	ALSN is an international nursing organization that values and respects the unique contributions of its members in advancing nursing leadership science. If ALSN were to develop a global leadership agenda for the next 3–5 years, what priorities would need to be included for it to be meaningful and useful in your leadership practice?

At the start of the session, the primary interviewer reviewed the informed consent information, explained the purpose of the study, and described how data would be collected, stored, and used. Participants were informed that participation was voluntary, that they could decline to respond to any question, and that they could withdraw from the discussion at any time without consequence. The interviewer also reminded participants of the importance of confidentiality among group members and asked that information shared during the session not be discussed outside the group. Verbal consent was obtained from each participant before the discussion began. The session lasted approximately 70 min and was video‐recorded with participants' knowledge and consent to support accurate transcription. Brief observational notes were also taken to capture context and group dynamics (Jayasekara 2012).

### 3.3 Data Management and Participant Privacy

Participant privacy was protected throughout data collection, transcription, analysis, and storage. Following the session, video recordings were transferred to a secure ALSN Box folder with access restricted to members of the research team. Transcripts were generated from the recordings and de‐identified prior to analysis; participants' names, country names, institutional affiliations, and any other potentially identifying details were removed or replaced with generic descriptors. Original recordings were retained only for the duration necessary to confirm transcript accuracy and then deleted in accordance with the approved data management plan. Only de‐identified transcripts were used during analysis. In presenting findings, participants are referred to by participant labels (P1, P2, and so on) rather than by name, and quotations have been reviewed to minimize the risk that any participant or country could be identified from the content reported.

### 3.4 Data Analysis

Data were analyzed using Braun and Clarke's (2006, 2021) six‐phase reflexive thematic analysis. Analysis began with repeated reading of the transcript and observational notes to support familiarization. Two team members independently coded the transcript and then met to compare interpretations and reconcile coding decisions. These two team members, together with two additional team members, subsequently reviewed and clustered related concepts and developed, refined, and named themes in relation to the study aims and relevant literature. Observational notes taken by additional research team members present during the focus group were reviewed against the transcript and the developed themes. Observer notes aligned with and supported the themes as constructed.

### 3.5 Trustworthiness

Trustworthiness was enhanced using established strategies for rigorous thematic analysis (Nowell et al. 2017). Credibility was supported through repeated engagement with the data, investigator triangulation, and peer debriefing. Dependability and confirmability were strengthened through adherence to Braun and Clarke's (2006, 2021) framework, an audit trail of analytic decisions, and a reflexive review of researcher perspectives. Transferability was supported through detailed descriptions of study procedures, analytic approach, and participant context, including attention to cultural and linguistic diversity (Halcomb et al. 2007; Jayasekara 2012).

### 3.6 Use of Generative AI

The authors acknowledge the use of Claude Opus 4.6 (accessed June 2026) to support reviewing, editing, and formatting the manuscript for clarity and flow. It also assisted with figure design, visual formatting, and colors. All AI‐generated suggestions were reviewed, revised, and approved by the authors, who take full responsibility for the accuracy and integrity of the work.

## 4 Results

**FIGURE 1:**
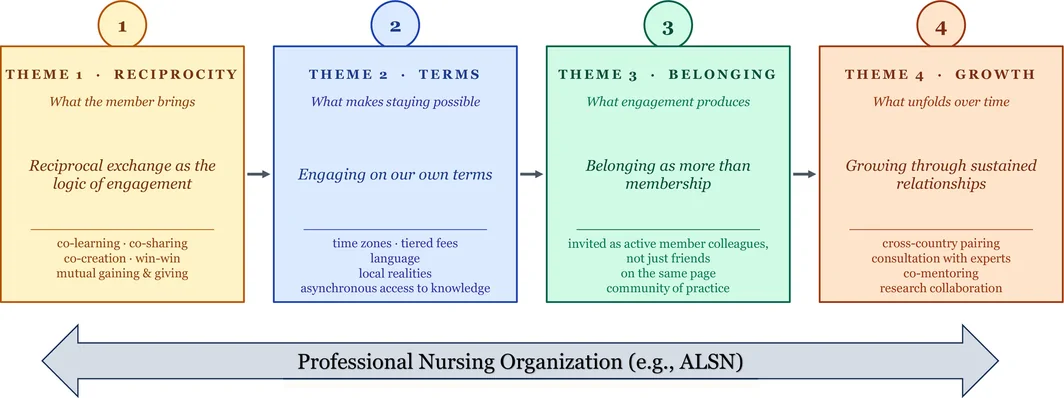
International nurse leaders' journeys of engagement, belonging, and growth.

Four themes were developed (see Figure 1) and are sequenced to reflect an international member journey: *reciprocal exchange as the logic of engagement*, *engaging on our own terms*, *belonging as more than membership*, and *growing through sustained international relationships*. A summary of the themes is presented in Table 2.

**TABLE 2:** Themes reflecting international ambassadors' journeys of engagement, belonging, and growth.

Theme	Description	Exemplar quotations
1. Reciprocal exchange as the logic of engagement	Members position themselves as contributors as well as recipients. Engagement is a mutual exchange in which knowledge, time, and recognition flow in both directions	“It's a win‐win. It's not only that they are benefiting or I'm benefiting.” (P1) “I'm seeking three things: co‐learning, co‐sharing, and co‐creation.” (P2)
2. Engaging on our own terms	Accessibility and context‐sensitivity are not amenities but conditions under which international members will engage	“If we have to pay fees, they have to take into consideration the economic status of the country.” (P1) “The time difference is a huge burden for me.” (P2)
3. Belonging as more than membership	Formal affiliation is not equivalent to belonging. Members experience belonging through invitations to participate in governance, peer convening, and visibility within the community	“I hope communication with you will give me the opportunity to have more colleagues, not just friends.” (P5) “Last year, I was invited to serve on the [regional organization] steering committee.” (P2)
4. Growing through sustained international relationships	Growth as nurse leaders occurs through ongoing cross‐border relationships, including mentoring, co‐mentoring, and consultation, rather than through time‐bound cohort programs alone	“If I'm paired intentionally with somebody not from my country, I can really facilitate that co‐mentoring and sharing.” (P3) “Our nurse leaders still need knowledge, experience, and some advice from U.S. leaders who have already passed through this issue before.” (P2)

### 4.1 Theme 1: Reciprocal Exchange as the Logic of Engagement

Participants did not describe membership in international organizations such as ALSN as something they had actively sought out, but they valued the opportunity when it emerged. For instance, P2 said, “I strongly believe that engaging with global organizations such as ALSN or Sigma Theta Tau will allow me to connect and learn from international leaders who come with different perspectives and experience. This is a meaningful opportunity that does not come easily.” However, participants emphasized that they wanted to enter the relationship as equals, bringing their own knowledge, time, and expertise. Reciprocity functions as the animating principle that members bring with them, the orienting logic through which they interpret every other feature of engagement. The exchange they describe is two‐way: members give knowledge, time, and experience, and they expect, in turn, to be recognized for what they bring. In this account, recognition is not a separate benefit; it is part of what makes the exchange reciprocal.

P1, describing their motivation to engage, said, “It's a win‐win. It's not only that they are benefiting or I'm benefiting. It's a win‐win situation.” When asked what would sustain engagement, P1 identified a “mutual relationship of gaining and giving,” emphasizing that members must not only receive but also be “sharing in giving our experience.” P1 grounded the reciprocal logic in an adult‐learning rationale: “adults do not engage until they find something they could benefit from. … We provide the knowledge, we provide the time, but we also expect something in return: visibility, connection, and knowledge generation through collaboration with colleagues from abroad.” P2 articulated the same logic in distinct language, condensing it into a triad: “I agree, and I'm seeking three things: co‐learning, co‐sharing, and co‐creation.” The triad captures the bidirectional movement, consistent with P1's description of engagement as “gaining and giving.” At the same time, P1's emphasis on “knowledge generation” and P2's emphasis on “co‐creation” support a generative dimension: members expect not only to exchange but to build something together. P2 positioned themselves as a contributor rather than a beneficiary, expressing willingness to “share my leadership experience and knowledge, if it could benefit others” and interest in “collectively creating a leadership project with other countries with different contexts and cultures.” The reciprocal logic also structured P3's and P4's accounts. P3 described their trajectory with Sigma Theta Tau as a shift from receiving to contributing, hoping for “a chance to contribute back … sometimes not necessarily from a leadership standpoint, but maybe from my [national] perspective.” P4 described their motivation to be a member of an organization: “Another part that motivates me to have a membership or to stay connected, which is also how I met [a current member of ALSN], is to make more impact.” For P4, reciprocity included visibility for one's contributions, not as personal credit but as the condition under which good practice could spread.

### 4.2 Theme 2: Engaging on Our Own Terms

Whether members can sustain engagement depends on whether the organization is structurally and culturally reachable in the conditions of their lives. Participants described accessibility and context‐sensitivity not as amenities but as the conditions under which continued membership is possible. Notably, they did not frame these as requests to the organization; they framed them as the terms on which they would engage.

P1 named the central condition in a single phrase: “accessibility and affordability.” For P1, accessibility meant “virtual meetings, like what you're doing now, at times that are appropriate for all of us,” and affordability meant that “if we have to pay fees, they have to take into consideration the economic status of the country.” P2 extended the same condition to temporal access: “the time difference is a huge burden for me,” asking for “an online learning platform, not just a virtual conference, but an accessible online learning platform I can access anytime.” P4 added, “not only fee‐wise, but also time‐wise,” asking for “resources you can actually and easily reach about leadership” that respected members' existing time constraints rather than adding to them.

To engage on their own terms, participants also spoke about the content of what was being offered, not just how it was being delivered. P1 insisted that any leadership development program must be “highly context‐sensitive,” considering “the local health system realities, the cultural dynamics, and the language of the community.” P3 extended the same reasoning into scholarship, warning against universalist assumptions: “what is common in certain demographics and certain geography doesn't mean it's common anywhere … it wouldn't be fair to do any type of research, leadership, or scholarship work if we were to assume everything's the same.” P4 articulated the gap between leadership theory and local practice from their own position, saying that “in my country we don't talk about leadership science as much” and that theoretical frameworks, when applied in practice settings, “have a lot to do with the culture, the style, and the people in it.” P2 made this point most concretely when discussing leadership frameworks and knowledge: “it cannot be like the theory; it has to be the reality.”

### 4.3 Theme 3: Belonging as More Than Membership

With reciprocity and engagement on their own terms in place, belonging becomes possible. Participants drew a clear distinction between formal membership in an organization and the felt experience of being part of a community of nurse leaders. Holding membership, paying dues, or appearing in a directory was not, in their accounts, equivalent to belonging. Belonging was described as meaningful inclusion: being invited to participate as active members, being recognized as peers, and finding themselves among colleagues with shared purpose and goals.

P5 had been formally and informally connected to international nursing organizations, attending [organization's] conference, presenting research findings there, and meeting senior leaders in person. Yet they stated plainly: “Unfortunately, I am not a member of [the organization].” Their hopes for ALSN were framed not as a request for services but as a longing for working relationships with peers: “I hope communication with you will give me the opportunity to have more colleagues, not just friends, and to have more research.”

The shift from formal membership to belonging was also described as arising from specific organizational actions. To be a member of an organization is to be listed as a member; to be invited onto its steering committee is to be recognized as someone whose voice the organization wants in its governance. P2 proudly shared, “last year, I was invited to serve on the [regional organization] steering committee.” The invitation, rather than membership itself, marked the moment of belonging.

Participants also pointed to structural practices through which belonging is cultivated over time. P3 described the [topic‐area group] they participated in, explaining the group's practice of hosting informal meet‐and‐greets whenever members happened to be at a conference and maintaining a shared WhatsApp chat that “worked really well” as a connective channel. Reflecting on what they gained, P3 said, “I'm meeting people on this call I would have never met at a conference or in any other way.”

Visibility was another way belonging could be cultivated. P1 emphasized the value of member profiles on the organization's website, describing their function in terms of internal knowability: ensuring that “every person who enters the website can see the names and interests of the individuals,” so the organization could “put into effect the people you are working with as ambassadors, and give them visibility to audiences who would like to work with them.” P2 echoed this with a proposal to “create a bank of international ambassadors and then set up some event that invites them to join. It can be a virtual conference, so that they can learn from each other.” Such infrastructure could transform a list of members into a recognizable community.

Taken together, these accounts show that belonging is distinct from formal membership and must be cultivated if members are to remain engaged. P4 captured a moment when this experience registered, noting that it was “good to know that we are actually on the same page.” What participants described wanting, and recognized when they had it, was the felt experience of being inside a community of nurse leaders who know one another, share aims, and treat each other as colleagues whose contributions matter.

### 4.4 Theme 4: Growing Through Sustained International Relationships

Participants described a form of professional growth that could not be delivered through content, events, or publications alone. It happened between people, over time, across borders. Whether called mentorship, consultation, collaboration, or co‐mentoring, participants were seeking the same underlying relationship: an ongoing cross‐border connection with leaders whose experience and presence shaped how they understood themselves and their work. They identified organizational structures, including intentional mentorship pairings, convening events, a speakers bureau, and advisory access, through which those relationships could form and hold.

P3 described what drew them into sustained membership in aspirational terms: “maintaining membership in these groups is a wonderful opportunity to develop a network of professionals I aspire to become more like.” When asked about priorities for ALSN's 3‐ to 5‐year agenda, they described what they hoped the mentorship program would become: “If I'm paired intentionally with somebody not from my country, I can really facilitate that co‐mentoring and sharing.” P3 also described the current program as worth extending to others: “I'm grateful, and I hope more people can have [access to the current program].”

P2 and P5 suggested that the organization create structures for a specific kind of relationship: sustained advisory access to nursing leaders from systems that had already navigated challenges their own countries were still facing. P2 said, “our nurse leaders still need knowledge, experience, and some advice from U.S. leaders who have already passed through this issue before.” P5 spoke to the same need regarding their country's ongoing nursing reform: “consultation with you in the process of transformation of our nursing would be a very helpful moment for us.” For both, the value was not in a one‐time transfer of information but in an ongoing advisory relationship that could accompany years of reform work.

P1 reframed one of ALSN's current structures, its speakers bureau, in relational terms: “the bank of names should not only include faculty from the U.S. It has to be international so that people involved can be provided with the experience of international interaction; this is what we call mentorship.” P2 extended this with a proposal for sustained peer learning across the ambassadors themselves: “if you can create a bank of international ambassadors, and then set up some event that invites them to join—it can be a virtual conference—you can collect all of them and they can learn from each other.” What transforms a directory into a vehicle of growth is the sustained interaction it enables.

Participants also identified emerging leadership challenges as areas where sustained international relationships could support growth. P2 spoke to newer domains as well, arguing that nursing leaders needed to engage with AI and digital technology not as recipients of tools designed by other professions but as designers: “we want AI that is created by our nurses, instead of any other profession creating something for us and saying it can replace you.” Workforce sustainability was the other priority they named, framed as an ongoing challenge that could be addressed through learning with leaders and systems further along on the same path.

P5 described the cumulative effect of relationships with nursing leaders over more than two decades of their country's nursing transformation: “You changed a lot in my country. You changed my life as well.” Relationships sustained over time, across borders, with leaders whose experience is trusted, do not just enhance growth as a nurse leader; in P5's case, they constituted it. Taken together, participants described a vision of nurse leader growth as something sustained by relationships, built and rebuilt over the years through ongoing connection, rather than delivered by any single program or event.

## 5 Discussion

This study explored the perspectives of nurse leaders enrolled in the ALSN International Ambassador Program to understand the motivations for and sustainment of engagement with an international nursing organization. Four themes were developed: reciprocal exchange as the logic of engagement, engaging on our own terms, belonging as more than membership, and growing through sustained international relationships. The themes describe a journey, beginning with the principles and values that international members bring to engagement and ending with the relational infrastructure through which they grow as nurse leaders. Each theme connects with and extends existing scholarship of professional nursing organizations, communities of practice, and global nursing mentorship.

The findings of the study expand current understanding of engagement and membership in international professional nursing organizations by positioning reciprocity as an essential design principle. The results highlight what one participant described as “co‐production,” whereby engagement in international organizations is mutual; members bring as much as they seek. This design principle shifts organizations' thinking from what to offer members to what infrastructure enables members to contribute in meaningful ways (Bazinski and Wilson 2023; Cline et al. 2019; Morin 2021). This new perspective extends traditional understandings of professional organizational membership as a way to grow personally and professionally, develop new knowledge, and receive support for creating change (Cline et al. 2019).

The findings suggest that international members in professional nursing organizations engage on particular terms that need to be recognized and addressed. Most importantly, organizations need to be sensitive to access (e.g., time zone issues, travel costs) and context (e.g., language, professional culture). These terms should be foundational to the organization's mission and international recruitment and engagement efforts. This study contributes to the existing literature on barriers to professional organization membership (Bazinski and Wilson 2023; Cline et al. 2019) by expanding beyond an emphasis on the benefits of networking, certification, and new knowledge to include an understanding of the terms of international members. Respecting these terms, in part, could be considered an equity issue. Future inquiry in this area could be strengthened by aligning the current findings with the Equity‐Centered Nursing Leadership Framework (ECNLF), a structured approach to advancing diversity in the nursing workforce (Ballout et al. 2026). The ECNLF provides strategies for dismantling systemic inequities and establishing sustainable leadership pathways that could be applied to building international membership in professional nursing organizations.

These results expand the literature on professional organization membership by identifying the importance of a felt sense of belonging among international members. This concept highlights that membership involves more than knowledge generation and networking opportunities. Members must feel that they belong (Wenger 1998). In the ALSN context, the authors' organizational experience suggested that formal membership had not always translated into a felt sense of voice or community among international members. Strategies to increase belonging may include peer support programs, mentorship initiatives, wellness activities, open communication, and technology‐enabled virtual connections (Frangieh et al. 2024).

The results indicate that sustained international engagement in professional organizations depends on relationships that foster growth rather than on time‐bound activities alone. Growth happens through ongoing cross‐border relationships embedded in the membership experience, not solely through one‐year cohort programs, events, or newsletters. Building relationships, working collaboratively on projects, and sharing ideas point to an unfolding process that matures over time.

Together, the four themes that emerged from this qualitative study can be understood as building blocks that support the sustainability of international membership: first, reciprocity honors what each member brings through co‐learning, co‐sharing, and co‐creating; second, recognition of international members' terms, including time zones, language, and resource availability, creates the conditions for access; third, belonging facilitates a community of practice and joint projects; and finally, sustained relationships support shared growth through cross‐country collaborations and co‐mentoring.

### 5.1 Implications for Professional Organizations and Researchers

The findings from this study, along with the post‐program assessment currently underway and to be published, can inform organizational program goals oriented around reciprocal contribution, equitable access, substantive belonging, and sustained cross‐border relationships. Efforts to foster sustained international relationships should not begin with relationship‐building alone; they first require structural conditions of access and a felt sense of belonging. Strategies to address the principles of reciprocity and the terms identified in this study include adding these priorities to the organization's mission and vision. Strategies to strengthen belonging might include global regional events framed around mutual learning, co‐producing solutions, and developing outputs that could be published and tested in multi‐site studies. To address access, organizations could develop a learning management system that includes content from the global community and a registry of members interested in collaboration to support cross‐country communication. Equity as a policy perspective further points to tiered fee structures that reflect economic diversity across regions, asynchronous learning platforms that reduce time zone burdens, and attention to language accessibility in materials and communication. Organizations seeking to extend meaningful international membership should invest in infrastructure for member onboarding that goes beyond formal joining, including intentional peer convening, invitations to participate in governance and committees, and structures that make international members visible and connected to one another. While Focus Group Question 4 asked participants about ALSN's 3–5 year strategic priorities, this analysis focused on cross‐cutting themes rather than extracting a discrete set of policy recommendations. A companion analysis of Question 4 responses is planned to directly inform ALSN's strategic priorities. Future quantitative or mixed‐methods research with a broader sample of international members could expand on these findings and test the patterns identified here across larger populations.

## 6 Limitations

This study has several limitations. First, the focus group included five of the 11 nurse leaders enrolled in the ALSN International Ambassador Program. While this sample provided rich insight into the experiences of international ambassadors who actively engage with the organization, it does not capture the perspectives of those who did not participate. The focus group was held as a single scheduled session, and time zones may have made attendance difficult for some ambassadors. Several ambassadors expressed regret that they could not attend because of scheduling conflicts. Second, the focus group represented a single point in time. It was conducted mid‐program, which may have influenced the findings; participants' perspectives may continue to evolve as the program develops and as members accumulate more sustained experience within it. Third, the use of a Zoom‐based focus group may have introduced limitations, including reduced ability to observe all non‐verbal cues in the online format (Willemsen et al. 2023). In addition, focus groups in any modality may be subject to social desirability bias because participants respond in the presence of others (Bergen and Labonté 2020). This risk may be heightened in the current study given participants' active enrollment in the Ambassador Program, which could incline them toward favorable rather than critical responses. However, participants voiced constructive critiques of current organizational structures, including the burden of time zones, the cost of participation, language barriers, and gaps in member induction, suggesting they felt able to speak candidly despite their program affiliation.

## 7 Conclusion

The four themes developed in this study are reciprocal exchange as the logic of engagement, engaging on our own terms, belonging as more than membership, and growing through sustained international relationships. International membership in professional nursing organizations can be enhanced by focusing on these four building blocks. Engagement is more likely to be sustained when organizations support reciprocity, meet international members on their own terms, foster belonging in the short‐ to medium‐term, and build sustained international relationships over time. The benefits of focusing on international engagement in nursing professional organizations include leveraging the collective expertise of nurses worldwide to address our most pressing problems, such as a shrinking nursing workforce and the increasing complexity of care.

## 8 Clinical Resources

1. Global Initiatives, Sigma Theta Tau International: https://www.sigmanursing.org/connect‐engage/our‐global‐impact.

2. Global Nursing Leadership Competency Framework, Sigma Theta Tau International: https://www.sigmanursing.org/global‐nursing‐leadership‐competency‐framework.

3. Global Nursing Leadership Institute, International Council of Nurses: https://www.icn.ch/how‐we‐do‐it/icn‐leadership‐centre/global‐nursing‐leadership‐institutetm‐gnli.

4. Nursing Now Challenge: https://www.nursingnow.org/.

5. Association for Leadership Science in Nursing: https://www.nursingleadershipscience.org/.

## Funding

The authors have nothing to report.

## Conflicts of Interest

The authors declare no conflicts of interest.

## Data Availability

Research data are not shared.
